# Reply to Letter to the Editor “Numerical Inconsistencies in ‘Physician-Staffed Ambulance Deployment: Comparative Response Time Analysis from a Slovak Pilot Project’”

**DOI:** 10.5811/westjem.68702

**Published:** 2026-10-18

**Authors:** Marian Sedlák, Tomas Petras, Imrich Berta, Gaston Ivanov, Jozef Karas

**Affiliations:** * Záchranná služba Košice, Košice, Slovak Republic; † Pavol Jozef Safarik University and Louis Pasteur University Hospital, Department of Trauma Surgery, Košice, Slovak Republic; ‡ Pavol Jozef Safarik University, Medical Education Centre, Košice, Slovak Republic; § Ministry of Health of the Slovak Republic, Institute for Healthcare Analyses, Bratislava, Slovak Republic; || Ministry of Health of the Slovak Republic, Health Section, Bratislava, Slovak Republic

Dear Editor:

We thank the correspondents for their careful reading of our study and for their constructive, collegial comments. We are grateful for the opportunity to correct errors that arose during data transcription and typesetting. We confirm that none of these errors affect the underlying analyses, results, or conclusions, and we address each point in turn below. We also welcome the interpretive observation regarding crew availability and clarify our position.

First, the correspondents are correct that the standard deviation of 34.63 minutes reported in Table 1 for the postintervention response interval of RLP Západ cannot arise from a sample bounded by the reported minimum of two minutes and the maximum of 51 minutes. This is a transcription error. The correct standard deviation, consistent with the other units and with the reported range, is 4.63 minutes; other data points are unaffected.

Second, we agree that the maxima of 12600 minutes (on-scene interval, RV Sever) and 19000 minutes (crew-availability interval, RV Sever) in Table 1 are implausible and reflect misplaced decimal points introduced during table preparation. The correct values are 126.00 minutes and 190.00 minutes, respectively. Importantly, the analyses were performed on the correctly scaled source data: the reported means (33.47 and 49.58 minutes) and standard deviations are computed from the true values and remain valid. Only the two tabulated maxima require correction.

Third, the 95% CI printed in Table 4 for the postintervention indicator, [−1.1013, −0.525], contains a typographical extra digit in the lower bound. The correct interval is [−1.013, −0.525], as consistently reported in the abstract, the population health research capsule, and the discussion sections, and as recovered from the coefficient and standard error (−0.7692 ± 1.96 × 0.124). Table 4 should be corrected accordingly.

Fourth, we confirm both observations concerning Table 4. (A) The intercept *t* value should read 46.9 (9.3295 / 0.199), not 49.6; the coefficient, standard error, and *P* value in that row are correct, and this is an isolated typesetting error. (B) For dispatch priority “M,” the lower bound of the 95% CI carries an incorrect sign; the correct interval is [−0.076, 0.386], which is consistent with the nonsignificant *P* value = .187. As the correspondents note, this covariate is nonsignificant and its interpretation is unchanged.

Fifth, we address the three items raised together. (A) The results text only transposes the district-level values; the correct pairing, as shown in Table 3, is Košice 2 (absolute reduction 2.15 minutes, r_e_ = 0.419) and Košice 3 (absolute reduction 2.64 minutes, r_e_ = 0.616). The sentence in the results section will be corrected to match Table 3. (B) The Table 3 footnote defining “r_e_” as “random effects” is an error; r_e_ denotes the rank-biserial correlation, as defined in the methods section, and the footnote will be corrected. (C) Regarding the sample sizes, the descriptive analyses in Table 1 include all 11,094 eligible cases, whereas the multivariable ordinary least squares regression was based on a more restricted analytic cohort. Although complete-case analysis is described in the methods section, the regression additionally incorporated specific model parameters, including month-fixed effects, dispatch priority, together with the time-related exclusion criteria. These additional criteria resulted in a complete analytic sample of *N* = 10,687. We agree that the eligibility and exclusion criteria for the multivariable analysis should have been stated more explicitly and will clarify these in the methods section.

Beyond these corrigible items, we appreciate the correspondents’ interpretive observation regarding crew availability. We acknowledge that the improvement was observed primarily in the unit transformed to the RV model, whereas the two remaining physician-staffed transport units showed the opposite trend. However, investigating the factors underlying these differences (including possible changes in case mix, workload, dispatch characteristics, or other operational factors) was beyond the scope of the study. Accordingly, we did not perform a formal system-wide analysis of crew availability and will revise the conclusion to distinguish the unit-level finding from broader system-level interpretations. As the correspondents also note, the primary outcome—response time—remains unaffected. We will revise the conclusion to distinguish the unit-level improvement in crew availability from broader system-level interpretations.

Finally, we remain committed to the principles of transparency and reproducibility. The underlying dataset and analytical materials are available upon reasonable request from the participating dispatch center, subject to the applicable governance and data-protection requirements. We welcome independent examination of our analyses and are currently planning to extend the study cohort, with the aim of repeating the analyses on a substantially larger dataset to evaluate the robustness and reproducibility of the present findings.

We again thank the correspondents for their rigorous and collegial review, which strengthens the accuracy of the published record.

Sincerely, on behalf of the authors’ team,

Marián Sedlák

